# Somatic Mutation of FAT Family Genes Implicated Superior Prognosis in Patients With Stomach Adenocarcinoma

**DOI:** 10.3389/fmed.2022.873836

**Published:** 2022-06-28

**Authors:** Qingjun Wang, Liang Cui, Pansong Li, Yuanyuan Wang

**Affiliations:** ^1^Department of Clinical Trial, The First Affiliated Hospital of Jinzhou Medical University, Jinzhou, China; ^2^GenePlus-Beijing Institute, Beijing, China

**Keywords:** stomach adenocarcinomas, FAT family, DNA damage repair, prognosis, somatic mutation, tumor microenvironment

## Abstract

FAT family genes encode protocadherin, which regulates tumor cell proliferation and migration. Although transcriptional levels of FAT family members had been reported in multiple malignant tumors, the association between mutation and prognosis of the FAT family in stomach adenocarcinoma (STAD) has not been investigated. Herein, we performed a multi-omics integrative bioinformatics analysis using genomic and mRNA expression data to explore the role of gene mutations across the FAT family on clinical outcomes of STAD. The results showed that FAT mutations occurred in 174 of 435 (40%) of the samples. Patients with FAT mutations possessed significantly better progression-free survival (*P* = 0.019) and overall survival (*P* = 0.034) than those with non-FAT mutations, and FAT mutations exhibited significantly higher tumor mutational burden (TMB) and microsatellite instability. Notably, FAT mutations had a greater effect on somatic single-nucleotide variation than copy number variation and resulted in more abundant DNA damage repair (DDR) mutations. Further investigation demonstrated that FAT mutations contributed to an inflammatory tumor microenvironment (TME), as indicated by significantly increased numbers of activated CD4 and CD8 T cells, and significantly decreased numbers of mast cell, plasmacytoid dendritic cell, type 2 T helper cell, and high expression of immune-promoting genes. Moreover, biological process antigen processing and presentation, DNA replication, and DDR-related pathways were significantly upregulated in patients with FAT mutations. Collectively, FAT mutations significantly improved the survival of patients with STAD by enhancing tumor immunogenicity (e.g., TMB and DDR mutations) and an inflamed TME, indicating that the FAT family might be a potential prognostic and therapeutic biomarker for STAD.

## Introduction

Gastric cancer (GC) is the fifth most frequently diagnosed cancer and the third leading cause of cancer death in the world, resulting in over 1,000,000 new cases and an estimated 783,000 deaths in 2018 (1). Stomach adenocarcinoma (STAD) is the most common form of GC. Currently, surgical resection remains the most feasible and complete cure for patients with STAD (2). The 5-year survival rate is fairly high in patients with STAD with an early diagnosis undergoing surgery, and the advanced patients usually have a dismal 5-year survival rate (3, 4). Unfortunately, the majority of patients with STAD worldwide except Japan and South Korea are first clinically diagnosed at an advanced stage, resulting in a poor overall prognosis, which might be attributed to multiple factors such as clinical, histopathological, and genetic differences (5). Actually, clinical prognostic factors, such as clinical symptoms and tumor stage, have limited predictive value for STAD treatment (6). Consequently, it is crucial to explore the pathogenesis and prognosis biomarkers of STAD.

To date, high-throughput sequencing has been used to determine individual genomic mutations. An increasing body of studies has shown that exploring potential genetic alterations involved in the cancer initiation and progression can identify clinically important biomarkers and potential therapeutic targets. Previous studies have found that the high expression of the genes *FN1*, *SERPINE1*, *SPARC*, *ANKRD33*, *OGN*, *JAM2*, *RERG*, *OLFML2B*, *ADAMTS1*, *DNER*, *LHCGR*, *NLRP14*, *OR4N2*, *PSG6*, *TTC29*, and *ZNF568* significantly predicted a poor prognosis of STAD (7–10). Moreover, although the molecular characterization of mutations in STAD has also been reported (11, 12), few studies have explored the connection between somatic mutations and STAD survival. Only a small percentage of research has shown that mutations of *BRCA2*, *MUC16*, and *DNAH* are associated with remarkably better survival outcomes (11, 13, 14). Hence, the somatic mutation events of STAD and their clinical effects deserve more attention.

The human FAT genes encode large transmembrane proteins with Cadherin repeats, epidermal growth factor (EGF)-like domains, and Laminin G-like domains, which frequently mutated across multiple cancer types (15, 16). At present, four cancer-related FAT variants had been reported. For instance, *FAT1* was considered a tumor suppressor gene or oncogene depending on the cancer types. Overexpressed *FAT1* inhibited tumorigenesis in esophageal squamous cell carcinoma (17), breast cancer (18), and head and neck squamous cell carcinoma (19), but promoted tumorigenesis in acute lymphoblastic leukemia (20), colorectal cancer (CRC) (21), GC (22), and hepatocellular carcinoma (23). The high expression of *FAT2* was significantly associated with poor prognosis in GC (24, 25), breast cancer (26), squamous cell carcinoma (27), and CRC (28). *FAT3* mutation or high expression was significantly correlated with poor prognosis in esophageal cancer (29) and patients with triple-negative breast cancer (30). Repression of *FAT4* expression was associated with an unfavorable prognosis of GC (31), CRC (32), and patients with triple-negative breast cancer (33). Collectively, these studies mainly focused on the mRNA levels of FAT family members, and there is no specific report on whether their mutations affect the development and prognosis of STAD.

In this study, we screened genes that had a remarkable effect on survival using STAD data from the TCGA database. Interestingly, each member of the FAT family could prolong progression-free survival (PFS) or overall survival (OS). Since the homologs of mutated genes can induce gene functional compensation (34), we explored the effect of the whole FAT gene family on STAD. The results showed that FAT mutations were correlated with better prognosis and attributed to an inflammatory tumor microenvironment (TME). Overall, FAT cadherins might be used as potential biomarkers and novel therapeutic targets for patients with STAD.

## Materials and Methods

### Data Acquisition

In this study, we used cBioPortal to download clinical and mutational information about patients with STAD from The Cancer Genome Atlas PanCancer study^1^, of whom 435 patients obtained somatic mutations and clinical data, 433 patients had analyzable copy number variation (CNV) data, and 408 patients possessed mRNA expression data (RSEM format).

### Tumor Mutational Burden and Microsatellite Instability Calculation

We analyzed the whole-exon sequencing data from the 435 primary STAD samples in the TCGA PanCancer study and calculated the mutation frequency in terms of the total number of non-synonymous mutations, including single-nucleotide substitutions (SNVs) and insertion–deletion (indel) mutations. Tumor mutational burden (TMB) was defined as the number of non-synonymous mutations detected at > 1% allele frequency in the coding region of the genome. We determined MSIsensor score > 4% as microsatellite instability (MSI), and MSIsensor score < 4% as microsatellite stability (MSS).

### Somatic Copy Number Variation Analysis

GISTIC version 2.0 was used to identify significantly amplified and deleted regions in this study. The genome doubling (GD) and ploidy data were determined by a previous study (35). The genomic instability index (GII) was calculated as the total length of copy number gain plus loss region in each sample divided by the genome length (36). Chromosome arms were labeled as “altered” in each group if GISTIC *q* < 0.1. To identify arm and focal-level CNV differences between the two groups, Fisher’s exact test was used for gains and losses, respectively, and a significant difference was determined as *P* < 0.05.

### DNA Damage Repair Pathway Enrichment

A DNA damage repair (DDR) gene list including 275 genes was collected from previously published research (37), of which 207 genes constitute eight canonical DDR pathways, namely, base excision repair (BER), nucleotide excision repair (NER), mismatch repair (MMR), the Fanconi anemia (FA), homologous recombination (HR), non-homologous end joining (NHEJ), direct damage reversal/repair (DR), and translesion synthesis (TLS) (38). The mutational count of pathways was obtained by calculating the total number of samples with at least one alteration in the corresponding pathway. Fisher’s exact test was used to reveal the potential differences in DDR pathways between FAT wild-type and mutant samples. The complete gene list is presented in Supplementary Table 1.

### Somatic Mutational Signature Analysis

DeconstructSigs package (version 1.8.0) was used to identify mutational signatures within a single tumor sample based on a negative matrix factorization (NMF) algorithm (39), which relies on the Bioconductor library BSgenome.Hsapiens.UCSC.hg19 to obtain mutational context information. The unique combination of mutation types in STAD samples with or without FAT mutations was constructed, and the mutational process was generated by COSMIC mutational signatures (version 2.0).

### Tumor Infiltrating Lymphocyte Cell Analysis

The mRNA expression data were transformed by log_2_ (RSEM + 1) for the single sample gene set enrichment analysis (ssGSEA) (40) to determine the infiltration level of 28 immune cell types by the “GSVA” R package (version 1.36.3). Marker genes for each immune cell type and immune-related genes and their functional classifications were obtained from the article published (41). Genes related to the antigen presentation, cell adhesion, chemokine, immunostimulator, and immunoinhibitor were collected from previous studies (41–43).

### Gene Set Enrichment Analysis

A total of 408 samples with gene expression profiles were partitioned into two groups according to the mutation status of FAT family genes. The RSEM values were rounded as input data. R package “DESeq2” was used to determine Fold Change from gene expression data between the two groups, and then all log2FoldChange values were used as input to the “clusterProfiler” R package to perform GSEA (44). The KEGG gene sets (version 7.4) were obtained from the MSigDB database^2^. The terms *q* < 0.05 were considered significant.

### Statistical Analysis

Survival analysis was performed using Kaplan–Meier curves, and the *P*-value was determined with the log-rank test. Fisher’s exact test was used to detect the proportion of mutually exclusive or co-occurring gene events between the two groups. The difference in continuous variables between the two groups was examined by the Wilcoxon method. Univariate Cox regression was used to assess the association between different variables and PFS or OS, and the results were presented as HRs and their 95% confidence intervals (CIs). All statistical tests were two-sided, and the result with *P* < 0.05 was considered statistically significant. All analyses and figure drawing were performed or generated using R version 4.0.3.

## Results

### The Characteristics of FAT Family Members in Stomach Adenocarcinoma

**FIGURE 1:**
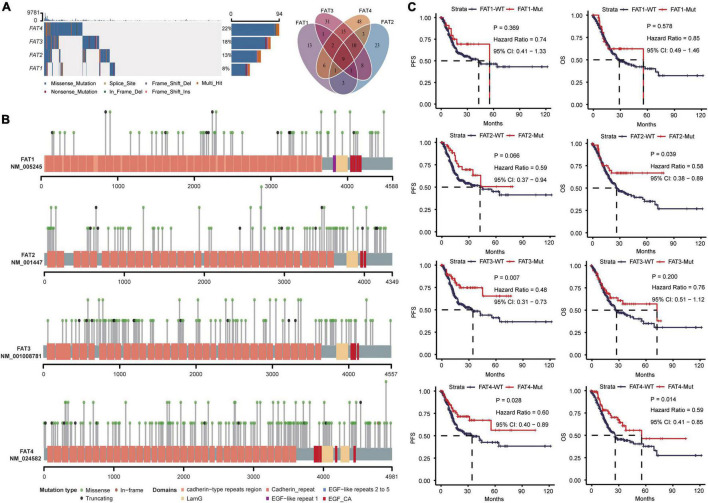
Mutations of FAT family members and its relationship with prognosis. **(A)** The mutational landscape of four FAT genes is showed (left). The Venn diagram showing the co-mutation numbers among different FAT members (right). **(B)** Display of protein functional domains and mutation sites of FAT family members in this study. **(C)** Kaplan-Meier analysis of progression-free survival (PFS) and overall survival (OS) in STAD patients with *FAT1*, *FAT2*, *FAT3*, or *FAT4* mutations, respectively.

A total of 435 TCGA STAD cases were identified, including 147,304 mutations in the exon. These mutations were in 17,566 genes, in which *TTN* (54%) was the most frequently mutated gene, and *FAT4* (22%) and *FAT3* (18%) mutations occurred in the top 20 mutated genes (Supplementary Figure 1). Other members of the FAT family had mutations with a frequency of 8% *FAT1* and 13% *FAT2*, and mutations in these four genes exhibited complex co-occurrence characteristics (Figure 1A). As shown in Figure 1B, FAT family genes all contain extracellular Cadherin repeats, EGF-like domains, and Laminin G-like domain, and multiple mutations occurred in the Cadherin repeats region, suggesting these FAT genes might play similar roles in STAD. We further explored the relationship between FAT family members and STAD prognosis and observed that *FAT2*, *FAT3*, and *FAT4* remarkably improved patient’s survival. Meanwhile, the same trend was also found in *FAT1* mutation, although the differences were not statistically significant (Figure 1C). Based on the above results, to better explore the influence of FAT family genes on STAD, we use the term “FAT mutations” to refer to the mutations of these four members of the FAT gene family in the subsequent analysis. Patients with any FAT family member mutation were divided into “FAT-Mut group,” and those without FAT mutations were “FAT-WT group.”

### FAT Mutations Are Associated With a Superior Prognosis in Stomach Adenocarcinoma

**FIGURE 2:**
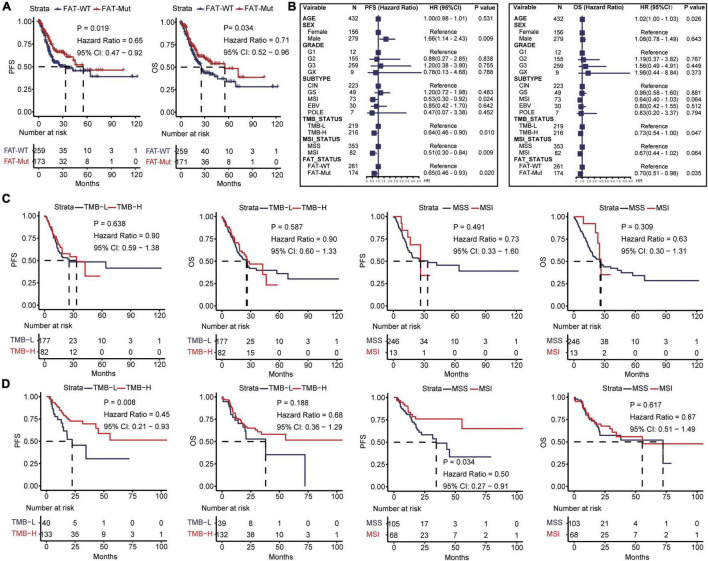
Impacts of FAT mutations on prognosis and clinical characteristics. **(A)** Kaplan-Meier analysis of PFS and OS in STAD patients with FAT mutations. **(B)** Univariate Cox regression analysis of age, gender, grade, subtype, TMB, MSI status, and FAT mutations. **(C)** Kaplan-Meier analysis of PFS and OS of TMB (the median TMB of all patients was 117 as cutoff) and MSI in FAT-WT patients. TMB, tumor mutational burden; MSS, microsatellite-stable; MSI, microsatellite instability. **(D)** Kaplan-Meier analysis of PFS and OS of TMB (the median TMB of all patients was 117 as cutoff) and MSI in FAT-Mut patients.

We further analyzed the connection between FAT mutations and outcomes in patients with STAD. It was found that FAT mutations had significantly better PFS (median: 55.4 vs. 33.5 month, HR = 0.65 [95% CI, 0.47–0.92], *P* = 0.019) and OS (median: 55.4 vs. 25.7 month, HR = 0.71 [95% CI, 0.52–0.96], *P* = 0.034) compared with those without mutations (Figure 2A); thus, FAT mutations were a positive prognostic factor for patients with STAD. Based on FAT status, we assessed the discrepancies in clinical characteristics between FAT-WT and FAT-Mut groups. No differences were discovered in sex and grade, whereas older age (*P* = 0.009), higher TMB (*P* < 0.001), and MSI (*P* < 0.001) were observed in the FAT-Mut group rather than the FAT-WT group (Table 1). In addition, univariate cox analysis showed that sex, TMB, and MSI statuses were related to PFS, while age and TMB were correlated with OS (Figure 2B). We further explored the association between FAT and TMB or MSI. In FAT-WT patients, high TMB (TMB-H) and MSI were unable to significantly prolong survival (Figure 2C). Nevertheless, TMB and MSI remained prognostic factors for PFS in patients with FAT mutations (Figure 2D).

**TABLE 1:** The clinical characteristics associated with FAT family genes.

Characteristics	FAT-WT (*N* = 261) No (%)	FAT-Mut (*N* = 174) No (%)	*P* value
**Histology**			
Stomach	261 (100)	174 (100)	
adenocarcinoma			
AGE			0.009
>60	162 (62.1)	130 (74.7)	
≤60	96 (36.8)	43 (24.7)	
Unkown	3 (1.1)	1 (0.6)	
SEX			0.476
Female	90 (34.5)	66 (37.9)	
Male	171 (65.5)	108 (62.1)	
GRADE			0.972
G1	7 (2.7)	5 (2.9)	
G2	95 (36.4)	60 (34.5)	
G3	154 (59.0)	105 (60.3)	
GX	5 (1.9)	4 (2.3)	
STAGE			0.074
I	27 (10.3)	29 (16.7)	
II	74 (28.4)	57 (32.8)	
III	123 (47.1)	64 (36.8)	
IV	28 (10.7)	15 (8.6)	
Unkown	9 (3.5)	9 (5.1)	
SUBTYPE			<0.001
CIN	159 (60.9)	64 (36.8)	
GS	35 (13.4)	14 (8.0)	
MSI	11 (4.2)	62 (35.6)	
EBV	21 (8.0)	9 (5.2)	
POLE	2 (0.8)	5 (2.9)	
Unkown	33 (12.7)	20 (11.5)	
TMB_STATUS			<0.001
TMB-L	179 (68.6)	40 (23.0)	
TMB-H	82 (31.4)	134 (77.0)	
MSI_STATUS			<0.001
MSS	248 (95.0)	105 (60.3)	
MSI	13 (5.0)	69 (39.7)	

*WT, wild-type; Mut, mutant; TMB, tumor mutational burden; MSS, microsatellite-stable; MSI, microsatellite instability. P-values were calculated using the Fisher’s exact test, and P-values < 0.05 were statistically significant.*

### FAT Mutations Result in More Abundant Single-Nucleotide Variation Events in Stomach Adenocarcinoma

**FIGURE 3:**
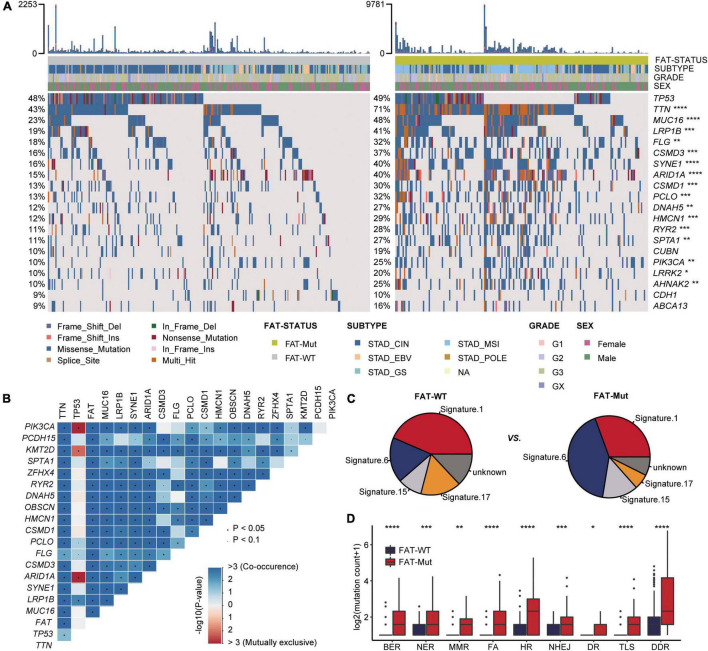
Somatic mutation characteristics associated with FAT mutations. **(A)** Top 20 frequently mutated genes in the FAT-WT (*n* = 261) and FAT-Mut (*n* = 174) groups. Genes are ranked by mutation frequency of FAT-WT patients. The proportion of mutated genes was tested by Fisher’s exact test. TCGA, The Cancer Genome Atlas (*P < 0.05; **P < 0.01; ***P < 0.001; and ****P < 0.0001; Fisher test). **(B)** Heatmap showing genes that are co-occurrent or mutual exclusive with mutated FAT in STAD. Red denotes mutual exclusivity, whereas blue indicates co-mutation. Fisher’s exact test was used to identify remarkable interactions, *P < 0.05. **(C)** The somatic mutational signature analysis. The proportion of total somatic substitutions in FAT-WT or FAT-Mut group contributed by each of the operative mutational signatures. **(D)** Comparison of DNA damage-related gene set alterations between FAT-WT and FAT-Mut patients with STAD. BER, base excision repair; NER, nucleotide excision repair; MMR, mismatch repair; FA, Fanconi anemia; HR, homologous recombination; NHEJ, non-homologous end joining; DR, direct repair; TLS, translesion synthesis (*P < 0.05; **P < 0.01; ***P < 0.001; and ****P < 0.0001; Wilcoxon test).

Based on somatic SNV data from 435 patients, the association between FAT mutations and exon mutation profiles in patients with STAD was further investigated. We found that the number of mutations was significantly higher in FAT-Mut patients than that in FAT-WT patients (Median: 246 vs. 85, *P* < 0.001, Wilcoxon test). Mutational landscape in patients with STAD showed that the FAT-Mut group had a higher mutation frequency than the FAT-WT group. The types of mutations were mainly missense and frameshift. Except for *TP53*, *CUBN*, *CDH1*, and *ABCA13*, the differences in the remaining top 20 genes were statistically significant (Figure 3A). Then, the mutually exclusive or co-occurring genes analysis demonstrated that *KMT2D* (12.6%), *ZFHX4* (12.9%), *TTN* (28.3%), *MUC16* (19.3%), *LRP1B* (16.3%), *CSMD3* (14.9%), *PCLO* (12.6%), *CSMD1* (12.2%), *HMCN1* (11.7%), *PIK3CA* (10.1%), *FLG* (12.9%), *OBSCN* (13.8%), *ARID1A* (16.1%), *SYNE1* (16.1%), *PCDH15* (11.5%), *RYR2* (11.3%), *SPTA1* (10.8%), and *DNAH5* (10.8%) were co-occurring events of FAT mutations, and no mutually exclusive genes associated with FAT mutations were found in this study (Figure 3B).

In addition, somatic mutational signature analysis was used to determine which internal boundary or external environmental factors were related to FAT mutations. We found that signature 1 (correlates with the age of cancer diagnosis), signature 6 (associated with defective DNA MMR), signature 15 (associated with defective DNA MMR), and signature 17 (the etiology remains unknown) were all identified regardless of the presence of FAT mutations, but signatures associated with DNA MMR were more abundant in the FAT-Mut group (Figure 3C). It is common knowledge that the DDR system is essential for maintaining genomic integrity, and gene mutations in the DDR will result in mutations/deletions in DNA that cannot be effectively corrected and the accumulation of incorrect DNA sequences, leading to tumor cell death. The number of gene mutations involved in several important pathways in the DDR system was significantly higher in the FAT-Mut group than in the FAT-WT group (DDR, *P* < 0.001) (Figure 3D), suggesting the FAT mutations might participate in the alterations of DDR-related pathways.

### Copy Number Variation Characteristics Based on FAT Status

**FIGURE 4:**
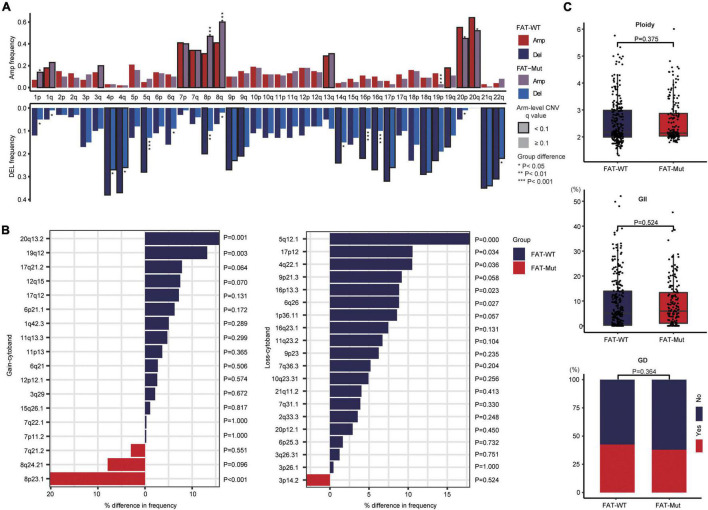
CNV analysis of FAT mutations. **(A)** Chromosome arm-level CNV frequencies in FAT-WT (*n* = 261) and FAT-Mut (*n* = 172) groups. Dark red (Amp: amplifications) and dark blue (Del: deletions) represent the FAT-WT group, while lighter colors represent the FAT-Mut group. Significantly altered arms in each group are highlighted with black borders; arms with significant group differences are denoted by asterisks (*P < 0.05; **P < 0.01; and ***P < 0.001; Fisher test). **(B)** Difference analysis in significantly loss or gain frequencies of cytobands contained in both FAT-Mut and FAT-WT groups, with *P*-values from Fisher’s exact test. **(C)** Comparison between FAT-WT and FAT-Mut groups according to copy number metrics: ploidy (*P* = 0.375), GII (*P* = 0.524), and GD (*P* = 0.364). *P*-values were calculated using the Wilcoxon test. GII, genomic instability index; GD, genome doubling.

Next, we undertook the somatic CNV analysis to search for genomic loci associated with FAT mutations. At the chromosomal level, the FAT-Mut group (*n* = 172) showed a lower degree of arm-level CNV than the FAT-WT group (*n* = 261), and such disparity occurred more in losses (Figure 4A). Besides, several focal CNVs around driver gene amplifications in *EGFR*, *ERBB2*, *MYC*, and *KRAS*, as well as deletions in *ARID1A*, *CDKN2A*, *SMAD4*, and *PTPRD* were found in FAT-WT and FAT-Mut groups (Supplementary Figure 2). Except for the cytoband existed in only one group, frequency differences of other cytobands between FAT-WT and FAT-Mut groups were compared (Figure 4B). 8p23.1 was the most significantly different cytobands, which was gained in the FAT-Mut group with a frequency of 48.3% compared to the FAT-WT group with 28.0% (*P* < 0.001). Hence, we speculated that a gain of 8p23.1 might be associated with FAT family gene mutations. Furthermore, ploidy, GD, and genome instability index (GII) were evaluated, all of which did not differ significantly between the FAT-WT and FAT-Mut groups (Figure 4C). Taken together, the effect of FAT mutations on CNV was limited.

### FAT Mutations Generate an Inflamed Tumor Microenvironment in Stomach Adenocarcinoma

**FIGURE 5:**
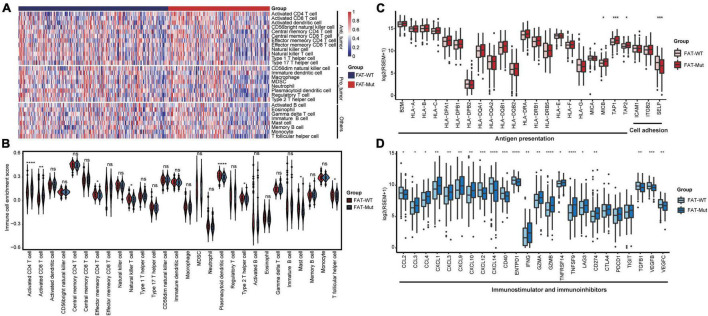
Immune infiltration analysis associated with FAT mutations. **(A)** The enrichment levels of 28 immune-related cells and types in the FAT-WT and FAT-Mut groups. **(B)** Comparisons of immune cells between FAT-Mut and FAT-WT patients with STAD. **(C,D)** The expression levels of immune-related genes, such as antigen presentation, cell adhesion, stimulation, and inhibition in FAT-Mut vs. FAT-WT patients with STAD. All *P*-values were calculated using the Wilcoxon test, *P < 0.05; **P < 0.01; ***P < 0.001; and ****P < 0.0001.

Herein, the ssGSEA algorithm was used to assess the differences in immune cell infiltration between the FAT-WT and FAT-Mut groups. The results demonstrated that the FAT-Mut group had an inflammatory TME, as indicated by significantly increased numbers of activated CD4 T cell and activated CD8 T cell and significantly decreased numbers of mast cell, plasmacytoid dendritic cell, and type 2 T helper cell (Figures 5A,B). Subsequently, immune-related gene expression profiles were analyzed in STAD patients with FAT mutations, the expression levels of genes related to activated immune cells (e.g., activated CD4 T cell and activated CD8 T cell) were significantly increased, and the expression levels of genes associated with suppressive immune cells (e.g., mast cell, plasmacytoid dendritic cell, and type 2 T helper cell) were remarkably reduced (Supplementary Figures 3A–E). The FAT-Mut group exhibited higher antigen presentation-related gene expression and lower expression of genes involved in cell adhesion (Figure 5C). The results of an analysis of stimulatory immune-related genes, such as chemokines (CCL3, CCL4, CXCL1, CXCL3, CXCL9, and CXCL10), cytokines IFNG, granzyme (GZMA and GZMB), tumor necrosis factor receptor superfamily (TNFRSF)-related genes TNFRSF14, and tumor necrosis factor (ligand) superfamily member TNFSF9, showed a significant upregulation in the FAT-Mut group (all *P*-values < 0.05). The expression of immune checkpoint genes, such as CD274 and LAG3, in FAT-Mut was significantly higher than that in FAT-WT, whereas CCL2, CXCL12, CXCL14, CD40, ENTPD1, TGFB1, and VEGF-related genes showed a lower expression in the FAT-Mut group (Figure 5D).

### Comparison of Transcriptomic Profiles Between FAT-WT and FAT-Mut Patients

**FIGURE 6:**
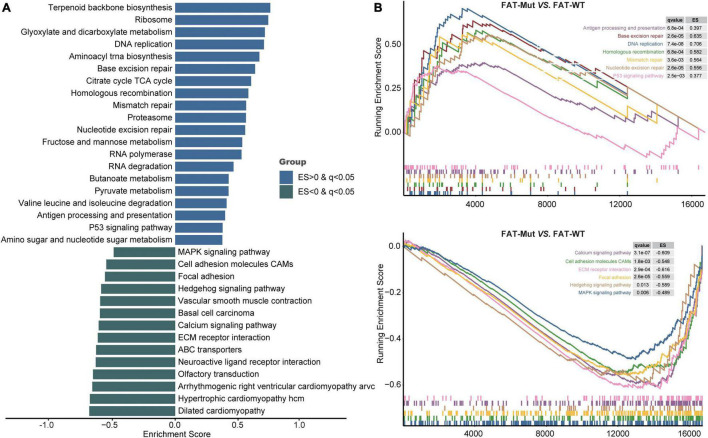
Transcriptomic analysis based on FAT status. **(A)** KEGG gene sets downloaded from the MSigDB, and GSEA was used to enrich pathways. Enrichment pathways with significant differences between FAT-Mut and FAT-WT groups are shown. The blue bar means ES is greater than 0; the green bar means ES is less than 0. MSigDB, Molecular Signatures Database; GSEA, gene set enrichment analysis; and ES, enrichment score. **(B)** Enrichment plots from GSEA. The pathways that are differentially enriched in FAT-Mut patients are shown at the top, and the pathways that are differentially enriched in FAT-WT patients are shown at the bottom.

To further investigate the biological processes affected by FAT family gene mutations in STAD, we performed GSEA to identify differential pathways between the FAT-WT and FAT-Mut groups. As shown in Figures 6A,B, the immune-related pathway antigen processing and presentation significantly upregulated in the FAT-Mut group. Moreover, P53 signaling pathway, metabolism-related pathways, DNA replication pathway, and DDR-related pathways, such as BER, MMR, NER, and HR, were also notably enriched in STAD patients with FAT family gene mutations (ES > 0 and *q* < 0.05). In contrast, adhesion-related pathways (e.g., cell adhesion molecules (CAMs) and focal adhesion), MAPK signal, Hedgehog signaling pathway, calcium signaling pathway, and ECM receptor interaction pathway were prominently enriched in the FAT-WT group (ES < 0 and *q* < 0.05). These results indicated that FAT family genes played an important role in the biological progression of STAD.

## Discussion

In this study, we determined differences in the somatic mutations, TME, immune-related gene expression profiles, and signaling pathways between FAT mutant and no-mutant groups using 435 STAD samples from the TCGA database. Compared with wild-type FAT, FAT mutations had a significant correlation with a better prognosis. Further investigation found that prolonged PFS and OS induced by FAT mutations might be associated with tumor immunogenicity (e.g., increased TMB, number of DDR-related gene mutations), activated CD4 and CD8 T cells, the expression of antigen processing and presentation-related genes, and significantly higher expression of immune-related genes. Moreover, the GSEA results showed that FAT mutations upregulated signaling pathways involved in antigen processing, DNA replication, and DDR-related pathways. In summary, these observations illustrated a possible mechanism to improve survival in STAD patients with FAT mutations. This is the first study to report the association between FAT family mutations and clinical outcomes of malignant tumors.

We found that *FAT2*, *FAT3*, and *FAT4* mutations significantly increased the survival time of patients with STAD, respectively. *FAT1* mutations had a similar trend, but there was no statistical difference, which may be due to the small sample size of *FAT1* mutations. Previous studies have reported that suppressing *FAT1* expression inhibited GC cell growth (22), the expression of *FAT2* in GC was significantly associated with lymph node and distant metastases and poor prognosis (24), and downregulation of *FAT4* expression in GC tissues was correlated with lymph node metastasis and poor prognosis (31). These results suggested that FAT family member mutations may retain similar functions and different mRNA expression patterns in STAD. FAT belongs to cadherin-related protein. It has been reported that cell adhesion facilitates tumor cell survival in the circulation and tumor cell extravasation (45). In our study, cell adhesion-related pathways (e.g., CAMs and focal adhesion) and cancer-promoting-related pathways (e.g., ECM, receptor interaction, Hedgehog signaling, calcium signaling, and MAPK signaling pathway) significantly enriched in FAT-WT patients, which might be associated with poor prognosis of FAT non-mutant patients.

FAT family genes frequently mutated across multiple malignant tumors (46). Several studies have detailed the biological functions of these proteins, such as Ena/VAPS-binding to FAT1 induces actin polymerization at lamellipodia and filopodia to promote cell migration (47), while Scribble-binding to FAT1 induces phosphorylation and functional inhibition of YAP1 to inhibit cell growth (16). FAT2 acts through the WAVE regulatory complex to drive collective cell migration during tissue rotation (48). FAT4 regulates the EMT and autophagy in colorectal cancer cells in part *via* the PI3K-AKT signaling axis (49). In this study, to explore whether the effect of FAT family mutations on STAD is specific, we analyzed the roles of FAT mutations in pan-cancer obtained from The Cancer Genome Atlas PanCancer study^3^. As shown in Supplementary Figure 4, FAT mutations significantly prolonged PFS and OS of patients with STAD or uterine corpus endometrial carcinoma and were detrimental to survival in patients with esophageal carcinoma, adrenocortical carcinoma, kidney renal papillary cell carcinoma, pancreatic adenocarcinoma, or pheochromocytoma and paraganglioma, suggesting FAT mutations were related to several tumors and might differentially affect tumor growth by regulating different biological processes. Recently, Feng et al. discover that FAT family genes are potential prognostic and immunological biomarkers and correlate with response to ICIs in non-small cell lung cancer (50), demonstrating that FAT family may also play roles in STAD immunotherapy. Another study reveals NFκB (RelA)/RelA/p65 as the transcriptional regulator of FAT1 gene in GBM cells (51), suggesting that transcriptional regulators might control the downstream signaling of FAT genes. As cellular factors are involved in the FAT family regulation of STAD, the deeper mechanism of influencing prognosis needs further experimental verification in future studies.

We further observed some molecular features associated with FAT mutations. Higher TMB was investigated in FAT-Mut patients rather than FAT-WT patients. A total of 4,306 significantly different SNV genes were identified between these two groups (Supplementary Table 2), and 18 genes co-occurred with FAT, whereas no genes that were mutually exclusive with FAT mutations were observed. Furthermore, patients with FAT mutations possessed more defective DNA MMR (dMMR) signature distribution and more abundant DDR-related gene mutations, as well as DDR-related signaling pathways. However, CNV analysis showed that FAT mutations were unable to affect GII levels and GD. At present, chromosomal instability (CIN) is considered to correlate with tumor metastasis (52), and whole genome-doubling (WGD) has been linked to increase tumor cell diversity, accelerate cancer genome evolution, and worse prognosis (53). Therefore, FAT mutations had a greater impact on SNV than CNV and did not result in alterations of chromosomal or large DNA sequences of STAD. Changes in SNV levels caused by FAT status may be one of the reasons that affect prognosis.

Microsatellite instability generated by dMMR gene mutations or epigenetic changes is considered to be one of the mechanisms of GC. Several studies had shown that GC patients with high MSI possessed its unique clinicopathological characteristics and good prognosis (54), and TMB was also significantly associated with DDR gene genotype in GC (55), which was consistent with our findings. Herein, to explore the relationship between FAT mutations and TMB or MSI, we evaluated the effect of TMB or MSI on the prognosis for FAT-WT and FAT-Mut patients and found that only when FAT mutated, TMB and MSI could stratify the benefits for patients, demonstrating that the effect of TMB and MSI on STAD prognosis depended on FAT mutations.

The interaction between tumor cells and infiltrating immune cells, fibroblasts, epithelial cells, vascular and lymphatic endothelial cells, as well as cytokines and chemokines constitutes the TME, which plays an important role in tumor development and progression (56). As mentioned earlier, patients with FAT mutations possessed higher TMB and more DDR mutations than those without FAT mutations, which may enhance tumor immunogenicity by generating more tumor neoantigen load (57). Comparing immune cells and immune-related gene expression across different FAT statuses, we found that in FAT mutant STAD, chemokines, such as CCL3, CCL4, CXCL1, CXCL3, CXCL9, and CXCL10, recruited and activated cytotoxic T lymphocytes in the tumor tissue to perform an antitumor effect. Antigen presentation-related genes, including MHC class I chain-related B (MICB), and transporter associated with antigen processing (TAP1 and TAP2) were significantly highly expressed in patients with FAT mutations, which contributed to the recognition of effector T cells and lymphocytes to tumor cells. Previous studies have reported that CD8 + TILs secrete granzyme, TNF, and perforin to exert cytotoxic function (58), and CD4 + TILs release IFNG and other cytokines (56), which is consistent with our study on the high expression of TNFRSF14, TNFSF9, GZMA, GZMB, and IFNG in the FAT-Mut group. Conversely, FAT-WT patients had suppressive TME with high expression of CCL2, CXCL12, CXCL14, CD40, ENTPD1, TGFB1, and VEGF; these factors have been confirmed to promote angiogenesis, invasion, and metastasis of tumor cells (59–64). Interestingly, immune checkpoints (e.g., LAG3 and CD274) showed an increased expression in patients with FAT mutations, suggesting that FAT mutations might be a cofactor in STAD immunotherapy.

There are still some limitations in our study. First, we only explored the possible roles of FAT mutations in STAD from the perspective of multi-omics integrative bioinformatics, and the deeper mechanism of influencing prognosis needs further experimental verification in the future. Second, whether FAT could be used as an independent risk predictor. In future studies, a large clinical cohort is required to verify the impact of FAT on prognosis. Third, which FAT family members play a more dominant role in the development of STAD and the interaction among FAT members will be explored in future studies. The current results should be considered preliminary for further mechanistic studies.

In conclusion, our study revealed that FAT mutations enhanced tumor immunogenicity (e.g., TMB and DDR mutations) and contributed to an inflammatory TME, thereby significantly improving the prognosis of patients with STAD, which might be a positive prognostic marker for STAD.

## Data Availability Statement

Publicly available datasets were analyzed in this study. This data can be found here: https://www.cbioportal.org/study/summary?id=stad_tcga_pan_can_atlas_2018.

## Author Contributions

YW conceived the study and designed the experiments. QW and LC performed the bioinformatic analysis and drafted the manuscript. PL provided insight in methodological approaches and analysis. YW supervised the study. All authors read and approved the final manuscript.

## Conflict of Interest

The authors declare that the research was conducted in the absence of any commercial or financial relationships that could be construed as a potential conflict of interest.

## Publisher’s Note

All claims expressed in this article are solely those of the authors and do not necessarily represent those of their affiliated organizations, or those of the publisher, the editors and the reviewers. Any product that may be evaluated in this article, or claim that may be made by its manufacturer, is not guaranteed or endorsed by the publisher.

## Footnotes

1
https://www.cbioportal.org/study/summary?id=stad_tcga_pan_can_atlas_2018

2
https://www.gsea-msigdb.org/gsea/msigdb/index.jsp

3
https://www.cbioportal.org/

## Supplementary Material

Supplementary Figure 1 Gene landscape of TCGA STAD patients. The top 20 genes in terms of mutation frequency genes are represented in the oncoprint.

Supplementary Figure 2 Enrichment of CNV cytoband in FAT-WT and FAT-Mut patients. The enrichment levels of all gain cytobands (top) and loss cytobands (bottom) in the STAD patients. Common driver genes associated with cytoband are labeled. The grouping and gene doubling information of each patient also is shown above the heatmap.

Supplementary Figure 3 The gene expression levels of immune cells related to FAT mutations. **(A,B)** The expression levels of genes related to activated immune cells: active CD4 T cell, and active CD8 T cell. **(C–E)** The expression levels of genes associated with suppressive immune cells: Mast cell, Plasmacytoid dendritic cell, and Type 2 T helper cell. Significance is calculated with Wilcoxon test, and indicated by asterisks. **P* < 0.05; ***P* < 0.01; ****P* < 0.001; and *****P* < 0.0001.

Supplementary Figure 4 The effect of FAT mutations on the prognosis of pan-cancer. Plot showing log_2_ (HR+1) (x axis) vs. −log_2_ (*P*-value) (y-axis) for a comparison of multiple tumors progression-free survival (PFS, left) and overall survival (OS, right) in FAT-Mut versus FAT-WT groups (*P*-value using log-rank test). The dotted lines mean *P* value = 0.05 or HR = 1. Cancers with *P* < 0.05 are colored in red. HR, hazard ratio; UCEC, Uterine Corpus Endometrial Carcinoma; STAD, Stomach adenocarcinoma; ESCA, Esophageal carcinoma; ACC, Adrenocortical carcinoma; KIRP, Kidney renal papillary cell carcinoma; LUAD, Lung adenocarcinoma; COADREAD, Colon and Rectal Cancer; CESC, Cervical squamous cell carcinoma and endocervical adenocarcinoma; THCA, Thyroid carcinoma; PCPG, Pheochromocytoma and Paraganglioma; HNSC, Head and Neck squamous cell carcinoma; MESO, Mesothelioma; DLBC, Lymphoid Neoplasm Diffuse Large B-cell Lymphoma; LGG, Brain Lower Grade Glioma; UVM, Uveal Melanoma; BLCA, Bladder Urothelial Carcinoma; OV, Ovarian serous cystadenocarcinoma; PAAD, Pancreatic adenocarcinoma; LIHC, Liver hepatocellular carcinoma; SKCM, Skin Cutaneous Melanoma; LUSC, Lung squamous cell carcinoma; GBM, Glioblastoma multiforme; SARC, Sarcoma; PRAD, Prostate adenocarcinoma; KIRC, Kidney renal clear cell carcinoma; UCS, Uterine Carcinosarcoma; KICH, Kidney Chromophobe; TGCT, Testicular Germ Cell Tumors; CHOL, Cholangiocarcinoma.

## Abbreviations

GC: gastric cancer
STAD: stomach adenocarcinomas
CRC: colorectal cancer
PFS: progression free survival
OS: overall survival
SNV: single-nucleotide variation
CNV: copy number variation
TMB: tumor mutational burden
MSI: microsatellite instability
MSS: microsatellite stability
DDR: DNA damage repair
BER: base excision repair
NER: nucleotide excision repair
MMR: mismatch repair
FA: Fanconi anemia
HR: homologous recombination
NHEJ: non-homologous end joining
DR: direct damage reversal/repair
TLS: translesion synthesis
ssGSEA: single sample gene set enrichment analysis
GSEA: gene set enrichment analysis
CIs: confidence intervals
CIN: chromosomal instability
WGD: whole genome-doubling.
