# Supplementary figures and images for: Somatic Mutation of FAT Family Genes Implicated Superior Prognosis in Patients With Stomach Adenocarcinoma
Source: Front Med (Lausanne). 2022 Jun 28;9:873836. doi: 10.3389/fmed.2022.873836 (PMC9273734; doi:10.3389/fmed.2022.873836)

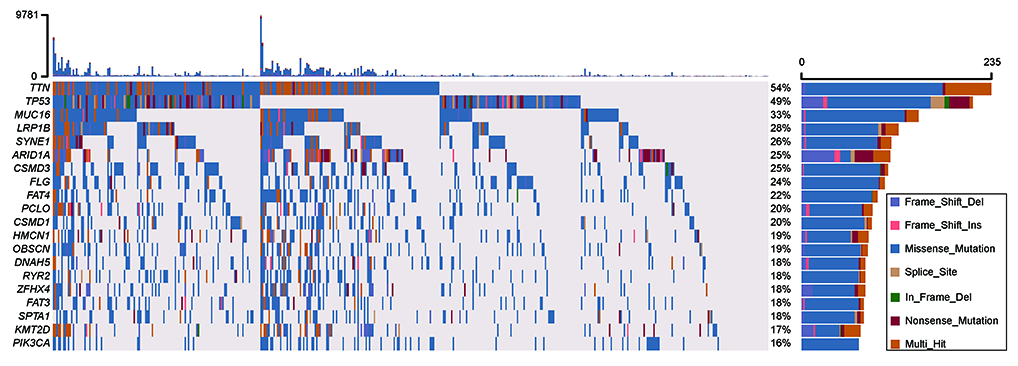

Supplement: Supplementary Figure 1 — Gene landscape of TCGA STAD patients. The top 20 genes in terms of mutation frequency genes are represented in the oncoprint. [file Image_1.tif]

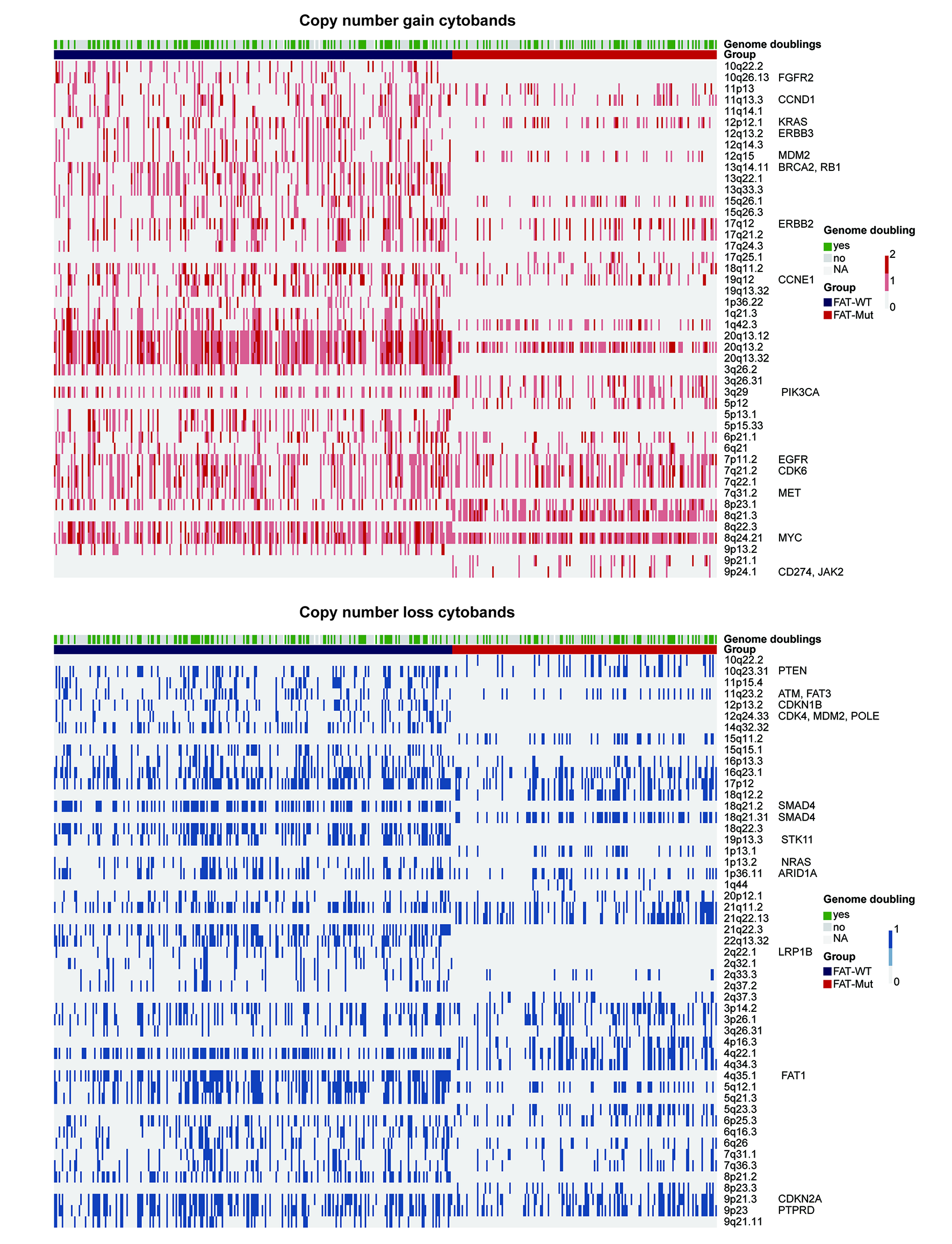

Supplement: Supplementary Figure 2 — Enrichment of CNV cytoband in FAT-WT and FAT-Mut patients. The enrichment levels of all gain cytobands (top) and loss cytobands (bottom) in the STAD patients. Common driver genes associated with cytoband are labeled. The grouping and gene doubling information of each patient also is shown above the heatmap. [file Image_2.tif]

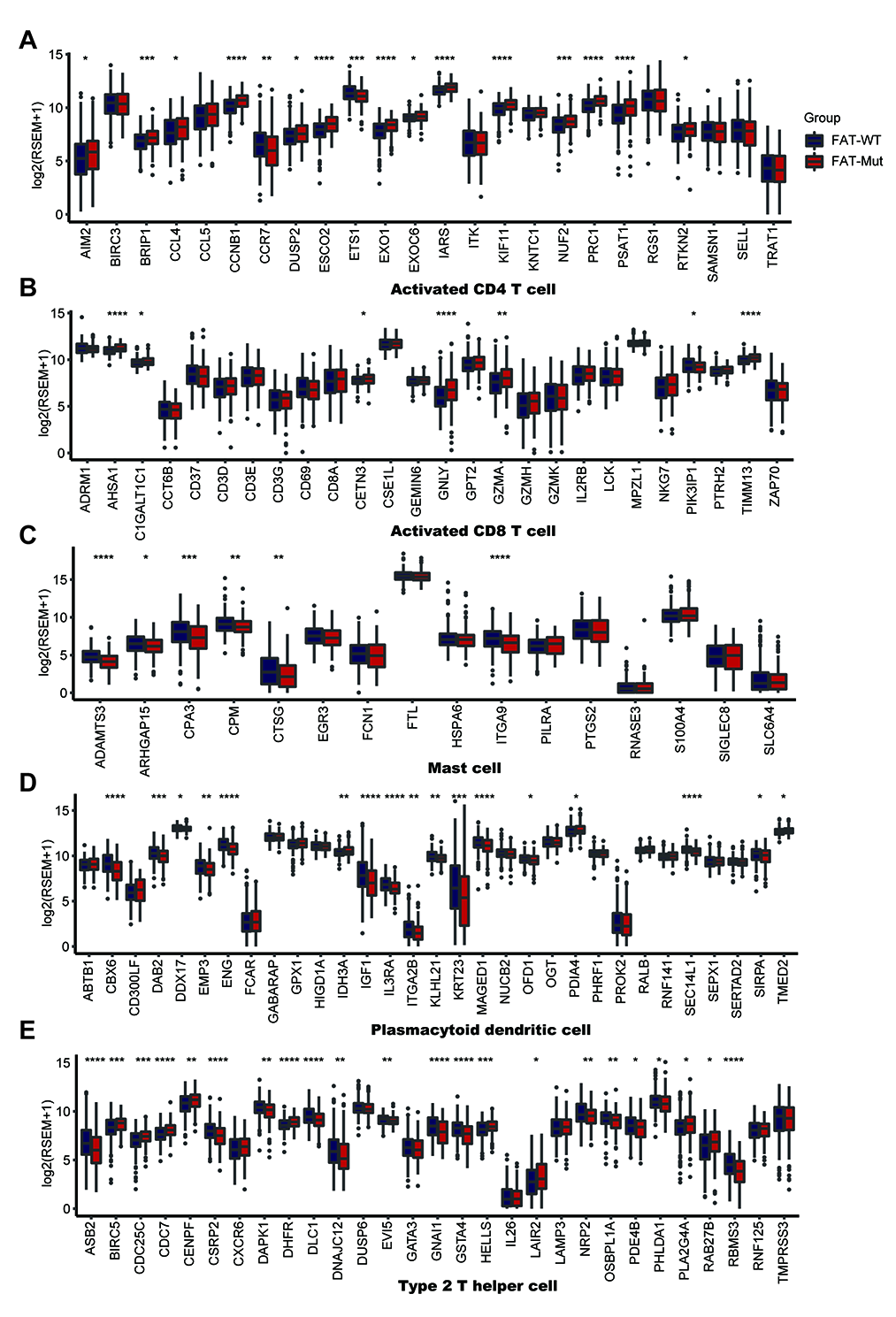

Supplement: Supplementary Figure 3 — The gene expression levels of immune cells related to FAT mutations. (A,B) The expression levels of genes related to activated immune cells: active CD4 T cell, and active CD8 T cell. (C–E) The expression levels of genes associated with suppressive immune cells: Mast cell, Plasmacytoid dendritic cell, and Type 2 T helper cell. Significance is calculated with Wilcoxon test, and indicated by asterisks. *P < 0.05; **P < 0.01; ***P < 0.001; and ****P < 0.0001. [file Image_3.tif]

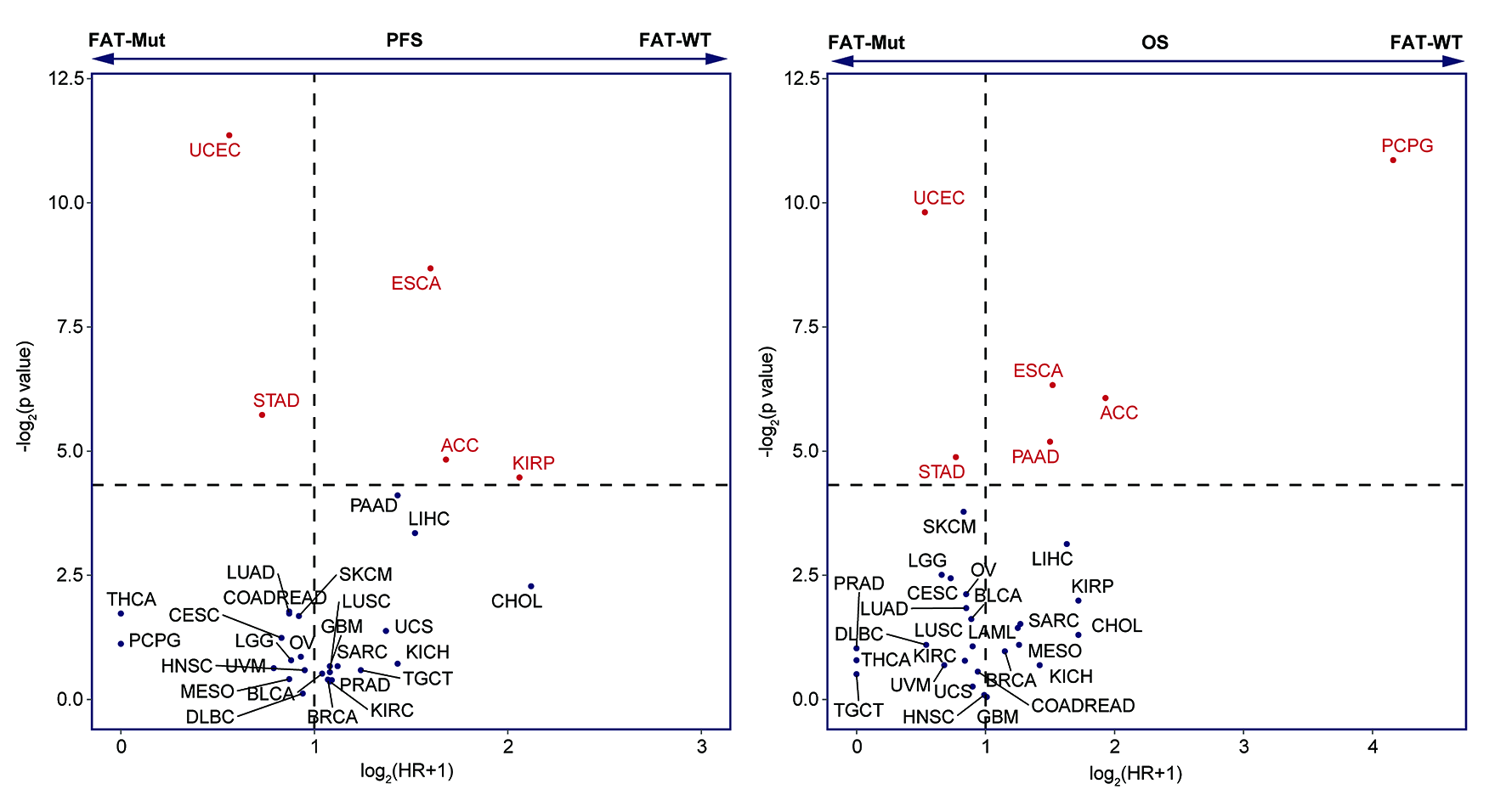

Supplement: Supplementary Figure 4 — The effect of FAT mutations on the prognosis of pan-cancer. Plot showing log2 (HR+1) (x axis) vs. −log2 (P-value) (y-axis) for a comparison of multiple tumors progression-free survival (PFS, left) and overall survival (OS, right) in FAT-Mut versus FAT-WT groups (P-value using log-rank test). The dotted lines mean P value = 0.05 or HR = 1. Cancers with P < 0.05 are colored in red. HR, hazard ratio; UCEC, Uterine Corpus Endometrial Carcinoma; STAD, Stomach adenocarcinoma; ESCA, Esophageal carcinoma; ACC, Adrenocortical carcinoma; KIRP, Kidney renal papillary cell carcinoma; LUAD, Lung adenocarcinoma; COADREAD, Colon and Rectal Cancer; CESC, Cervical squamous cell carcinoma and endocervical adenocarcinoma; THCA, Thyroid carcinoma; PCPG, Pheochromocytoma and Paraganglioma; HNSC, Head and Neck squamous cell carcinoma; MESO, Mesothelioma; DLBC, Lymphoid Neoplasm Diffuse Large B-cell Lymphoma; LGG, Brain Lower Grade Glioma; UVM, Uveal Melanoma; BLCA, Bladder Urothelial Carcinoma; OV, Ovarian serous cystadenocarcinoma; PAAD, Pancreatic adenocarcinoma; LIHC, Liver hepatocellular carcinoma; SKCM, Skin Cutaneous Melanoma; LUSC, Lung squamous cell carcinoma; GBM, Glioblastoma multiforme; SARC, Sarcoma; PRAD, Prostate adenocarcinoma; KIRC, Kidney renal clear cell carcinoma; UCS, Uterine Carcinosarcoma; KICH, Kidney Chromophobe; TGCT, Testicular Germ Cell Tumors; CHOL, Cholangiocarcinoma. [file Image_4.tif]
