# Supplementary material for: Somatic Mutation of FAT Family Genes Implicated Superior Prognosis in Patients With Stomach Adenocarcinoma
Source: Front Med (Lausanne). 2022 Jun 28;9:873836. doi: 10.3389/fmed.2022.873836 (PMC9273734; doi:10.3389/fmed.2022.873836)
Supplement: Supplementary file 5 [file Table_1.docx]

**Table S1. Annotation of 275 DDR genes to specific DNA damage repair pathways.**

| Pathway | Hugo_Symbol |
| --- | --- |
| Base Excision Repair (BER) | APLF, APTX, LIG1, LIG3, PARG, PARP1, PARP3, PNKP, POLB, POLL, XRCC1, HMGB1, PCNA, POLD1, POLD2, POLD3, POLD4, RFC1, RFC2, RFC3, RFC4, RFC5, ALKBH1, APEX1, APEX2, FEN1, HMGB2, MBD4, MPG, MUTYH, NEIL1, NEIL2, NEIL3, NTHL1, OGG1, PARP2, PARP4, POLE, POLE2, POLE3, POLE4, POLK, SMUG1, TDG, TDP1, UNG, WRN |
|  |  |
| Nucleotide Excision Repair (NER) | LIG1, PCNA, POLD1, POLD2, POLD3, POLD4, RFC1, RFC2, RFC3, RFC4, RFC5, RPA1, RPA2, RPA3, RPA4, CCNH, CDK7, CETN2, CUL3, CUL4A, CUL5, DDB1, DDB2, ERCC1, ERCC2, ERCC3, ERCC4, ERCC5, ERCC6, ERCC8, GTF2H1, GTF2H2, GTF2H3, GTF2H4, GTF2H5, MMS19, MNAT1, POLE, POLE2, POLE3, POLE4, RAD23A, RAD23B, RBX1, TCEA1, TCEB1, TCEB2, UVSSA, XAB2, XPA, XPC |
|  |  |
| Mismatch Repair (MMR) | LIG1, EXO1, HMGB1, MLH1, MLH3, MSH2, MSH3, MSH6, PCNA, PMS1, PMS2, POLD1, POLD2, POLD3, POLD4, RFC1, RFC2, RFC3, RFC4, RFC5, RPA1, RPA2, RPA3, RPA4 |
|  |  |
| Fanconi Anemia (FA) | XRCC2, APITD1, BARD1, BLM, BRCA1, BRCA2, BRE, BRIP1, ERCC1, ERCC4, FAAP100, FAAP24, FAAP20, FAN1, FANCA, FANCB, FANCC, FANCD2, FANCE, FANCF, FANCG, FANCI, FANCL, FANCM, HELQ, HES1, MAD2L2, PALB2, RAD51, RAD51C, RMI1, RMI2, SLX1A, SLX4, STRA13, TELO2, TOP3A, TOP3B, UBE2T, USP1, WDR48 |
|  |  |
| Homology-dependent recombination (HDR) | LIG1, MRE11A, NBN, PARG, PARP1, PARPBP, RAD50, TP53BP1, XRCC2, XRCC3, EXO1, PCNA, POLD1, POLD2, POLD3, POLD4, RFC1, RFC2, RFC3, RFC4, RFC5, RPA1, RPA2, RPA3, RPA4, BARD1, BLM, BRCA1, BRCA2, BRIP1, DMC1, DNA2, EID3, EME1, EME2, ERCC1, FANCM, FEN1, GEN1, HFM1, H2AFX, HELQ, INO80, KAT5, MUS81, NFATC2IP, NSMCE1, NSMCE2, NSMCE3, NSMCE4A, PALB2, PARP2, PAXIP1, POLH, POLQ, PPP4C, PPP4R1, PPP4R2, PPP4R4, RAD51, RAD51B, RAD51C, RAD51D, RAD52, RAD54B, RAD54L, RBBP8, RDM1, RECQL, RECQL4, RECQL5, RMI1, RMI2, RTEL1, SHFM1, SLX1A, SLX1B, SLX4, SMARCAD1, SMC5, SMC6, SPO11, SWSAP1, TOP3A, TOP3B, UIMC1, WRN, ZSWIM7 |
|  |  |
| Non-homologous End Joining (NHEJ) | DNTT, LIG4, MRE11A, NBN, NHEJ1, PARG, PARP1, PARP3, PNKP, POLB, POLL, POLM, PRKDC, RAD50, RNF168, RNF8, TP53BP1, XRCC4, XRCC5, XRCC6, DCLRE1C, FAM175A, RIF1 |
|  |  |
| Direct Repair (DR) | ASCC3, ALKBH2, ALKBH3, MGMT |
|  |  |
| Translesion Synthesis (TLS) | POLB, POLM, UBE2A, PCNA, HLTF, MAD2L2, POLH, POLI, POLK, POLN, POLQ, RAD18, REV1, REV3L, SHPRH, UBE2B, UBE2N, UBE2V2, USP1, WDR48 |
|  |  |
| DDR | APLF, APTX, LIG1, LIG3, PARG, PARP1, PARP3, PNKP, POLB, POLL, XRCC1, HMGB1, PCNA, POLD1, POLD2, POLD3, POLD4, RFC1, RFC2, RFC3, RFC4, RFC5, ALKBH1, APEX1, APEX2, FEN1, HMGB2, MBD4, MPG, MUTYH, NEIL1, NEIL2, NEIL3, NTHL1, OGG1, PARP2, PARP4, POLE, POLE2, POLE3, POLE4, POLK, SMUG1, TDG, TDP1, UNG, WRN, RPA1, RPA2, RPA3, RPA4, CCNH, CDK7, CETN2, CUL3, CUL4A, CUL5, DDB1, DDB2, ERCC1, ERCC2, ERCC3, ERCC4, ERCC5, ERCC6, ERCC8, GTF2H1, GTF2H2, GTF2H3, GTF2H4, GTF2H5, MMS19, MNAT1, RAD23A, RAD23B, RBX1, TCEA1, TCEB1, TCEB2, UVSSA, XAB2, XPA, XPC, EXO1, MLH1, MLH3, MSH2, MSH3, MSH6, PMS1, PMS2, XRCC2, APITD1, BARD1, BLM, BRCA1, BRCA2, BRE, BRIP1, FAAP100, FAAP24, FAAP20, FAN1, FANCA, FANCB, FANCC, FANCD2, FANCE, FANCF, FANCG, FANCI, FANCL, FANCM, HELQ, HES1, MAD2L2, PALB2, RAD51, RAD51C, RMI1, RMI2, SLX1A, SLX4, STRA13, TELO2, TOP3A, TOP3B, UBE2T, USP1, WDR48, MRE11A, NBN, PARPBP, RAD50, TP53BP1, XRCC3, DMC1, DNA2, EID3, EME1, EME2, GEN1, H2AFX, HFM1, INO80, KAT5, MUS81, NFATC2IP, NSMCE1, NSMCE2, NSMCE3, NSMCE4A, PAXIP1, POLH, POLQ, PPP4C, PPP4R1, PPP4R2, PPP4R4, RAD51B, RAD51D, RAD52, RAD54B, RAD54L, RBBP8, RDM1, RECQL, RECQL4, RECQL5, RTEL1, SHFM1, SLX1B, SMARCAD1, SMC5, SMC6, SPO11, SWSAP1, UIMC1, ZSWIM7, DNTT, LIG4, NHEJ1, POLM, PRKDC, RNF168, RNF8, XRCC4, XRCC5, XRCC6, DCLRE1C, FAM175A, RIF1, ASCC3, ALKBH2, ALKBH3, MGMT, UBE2A, HLTF, POLI, POLN, RAD18, REV1, REV3L, SHPRH, UBE2B, UBE2N, UBE2V2, NUDT1, NUDT15, NUDT18, RRM1, RRM2, ATM, ATR, ATRIP, ATRX, CHAF1A, CHEK1, CHEK2, CLK2, DCLRE1A, DCLRE1B, DUT, GADD45A, GADD45G, HUS1, MDC1, MPLKIP, MRPL40, NABP2, PER1, POLA1, POLG, PRPF19, RAD1, RAD17, RAD9A, RNMT, RRM2B, SETMAR, TOPBP1, TP53, TREX1, TREX2, TYMS, PTEN, TDP2, ENDOV, SPRTN, RNF4, SMARCA4, IDH1, SOX4, WEE1, RAD9B, AEN, PLK3, EXO5, CDC5L, BCAS2, PLRG1, YWHAB, YWHAG, YWHAE, CDC25A, CDC25B, CDC25C, BABAM1, BRCC3, TTK, SMARCC1, SWI5, MORF4L1, RNF169, HERC2 |
|  |  |
